# Sequencing chemotherapy and radiotherapy in locoregional advanced breast cancer patients after mastectomy – a retrospective analysis

**DOI:** 10.1186/1471-2407-8-114

**Published:** 2008-04-23

**Authors:** Marc D Piroth, Michael Pinkawa, Bernd Gagel, Sven Stanzel, Branka Asadpour, Michael J Eble

**Affiliations:** 1 Department of Radiation Oncology, RWTH Aachen University Hospital, Aachen, Germany; 2 Institute of Medical Statistics, RWTH Aachen University Hospital, Aachen, Germany

## Abstract

**Background:**

Combined chemo- and radiotherapy are established in breast cancer treatment. Chemotherapy is recommended prior to radiotherapy but decisive data on the optimal sequence are rare. This retrospective analysis aimed to assess the role of sequencing in patients after mastectomy because of advanced locoregional disease.

**Methods:**

A total of 212 eligible patients had a stage III breast cancer and had adjuvant chemotherapy and radiotherapy after mastectomy and axillary dissection between 1996 and 2004. According to concerted multi-modality treatment strategies 86 patients were treated sequentially (chemotherapy followed by radiotherapy) (SEQgroup), 70 patients had a sandwich treatment (SW-group) and 56 patients had simultaneous chemoradiation (SIM-group) during that time period. Radiotherapy comprised the thoracic wall and/or regional lymph nodes. The total dose was 45–50.4 Gray. As simultaneous chemoradiation CMF was given in 95.4% of patients while in sequential or sandwich application in 86% and 87.1% of patients an anthracycline-based chemotherapy was given.

**Results:**

Concerning the parameters nodal involvement, lymphovascular invasion, extracapsular spread and extension of the irradiated region the three treatment groups were significantly imbalanced. The other parameters, e.g. age, pathological tumor stage, grading and receptor status were homogeneously distributed. Looking on those two groups with an equally effective chemotherapy (EC, FEC), the SEQ- and SW-group, the sole imbalance was the extension of LVI (57.1 vs. 25.6%, p < 0.0001).

5-year overall- and disease free survival were 53.2%/56%, 38.1%/32% and 64.2%/50%, for the sequential, sandwich and simultaneous regime, respectively, which differed significantly in the univariate analysis (p = 0.04 and p = 0.03, log-rank test). Also the 5-year locoregional or distant recurrence free survival showed no significant differences according to the sequence of chemo- and radiotherapy. In the multivariate analyses the sequence had no independent impact on overall survival (p = 0.2) or disease free survival (p = 0.4). The toxicity, whether acute nor late, showed no significant differences in the three groups. The grade III/IV acute side effects were 3.6%, 0% and 3.5% for the SIM-, SW- and SEQ-group. By tendency the SIM regime had more late side effects.

**Conclusion:**

No clear advantage can be stated for any radio- and chemotherapy sequence in breast cancer therapy so far. This could be confirmed in our retrospective analysis in high-risk patients after mastectomy. The sequential approach is recommended according to current guidelines considering a lower toxicity.

## Background

Adjuvant chemotherapy and radiotherapy were established in the multidisciplinary treatment of breast cancer. Generally, radiotherapy is used after completion of adjuvant chemotherapy but decisive data for a scientifically based decision on the optimal sequence are not known. The majority of published data are related to early breast cancer (stage I-II). The only existing randomized study questioning the optimal sequencing of radio- and chemotherapy in early breast cancer found no substantial differences in locoregional or distant recurrence between the treatment arms [1,2]. But in patients with locoregional advanced breast cancer (stage III) surgically treated with mastectomy and axillary dissection, no data on the optimal chemo- and radiotherapy sequence exists. The objective of our retrospective study was to evaluate the clinical outcome of our female patients with locoregional advanced breast cancer after mastectomy and adjuvant radiotherapy, with a specific interest on the role of sequencing chemo- and radiotherapy.

## Methods

In the database of the Department of Radiation Oncology, 343 female patients were found to have mastectomy with axillary dissection and adjuvant radiotherapy, following the diagnosis of unilateral locoregional advanced invasive breast cancer in the time period from January 1996 to June 2004. Eligible criteria for the retrospective analysis were a stage III breast cancer treated with mastectomy and adjuvant chemo- and radiotherapy. A cohort of 212 patients fulfilled the criteria and was included in our analysis.

The data presented in this analysis were acquired retrospectively and anonymously. According to the regulations of the German Medical Association no official approval by the local ethics committee was necessary. All patients had a primary locoregional advanced disease, characterized by a pT3/pT4 tumor in 41.9% of patients, an involvement of at least four axillary lymph nodes (60.8% of patients) and evidence of multicentricity in 36.3% of patients. An inflammatory disease was seen in 3.4% of the included patients. The surgical procedure was performed in the Department of Gynaecology and Obstetrics at the University Hospital Aachen and at corresponding experienced Departments of Gynaecology from four regional hospitals. In periodical conferences, all patients were discussed and the therapeutic approach determined. After surgery all patients were irradiated in the Dept. of Radiation Oncology, RWTH Aachen University Hospital.

Surgery included modified radical mastectomy or, in a few patients simple mastectomy and in all patients axillary lymph node dissection (usually levels I and II). An immediate reexcision was performed in 12 patients (5.7%) with microscopically incomplete resection. Finally the pathological margin was negative in 92.9% of the patients.

Following surgery, all women received adjuvant chemotherapy and adjuvant external beam radiotherapy.

The patient age ranged from 34 to 92 (median 64) years.

The patients were grouped according to the sequence of chemotherapy and radiotherapy. A group of 86 patients (40.6%) was treated sequentially (SEQ-group) which means that radiotherapy was applied after finishing the last chemotherapy cycle. Seventy patients (33.9%) were treated in a sandwich scheme (SW-group) which means that 2–4 chemotherapy cycles were given prior to radiotherapy followed by 2–4 further chemotherapy cycles, and 56 patients (26.4%) were treated with simultaneous chemo- and radiotherapy (SIM-group) in the years 1996–1999. In the SEQ- and the SW-group most of the patients (86% and 87.1%) had an antracycline-based chemotherapy regime (EC or FEC) while in the SIM-group a CMF chemotherapy was given in 95.4% of patients.

From the patho-histological examination 158 (74.5%) and 35 (16.5%) patients had ductal-invasive and lobular-invasive breast cancer, respectively. Nineteen (9%) patients had a rare histology (medullary (n = 8), papillary (n = 4), tubular (n = 2), other (n = 5)).

A total of 158 patients (74.5%) had positive estrogen and/or progesterone receptors and usually received an adjuvant antihormonal therapy.

Radiotherapy was performed typically. The ipsilateral supra- and infraclavicular lymph nodes and the axilla (SIA) were irradiated with anterior-posterior (ap)- or anterior-posterior/posterior-anterior (ap/pa)- fields with 6–10 megaelectron volt (MeV) photons. The thoracic wall (THW) and the ipsilateral parasternal lymph nodes were irradiated with individually shaped electron fields with 4–12 and 10–15 MeV. The total dose in all fields was 45–50.4 Gray (Gy) in daily fractions of 1.8–2 Gy.

All patients gave written informed consent to surgery, radiotherapy and chemotherapy, separately.

The radiotherapy induced acute and late toxicity was analyzed according to the RTOG- (Radiation Therapy Oncology Group) scoring system related to the radiotherapy specific side effects as skin erythema, teleangiectasia, hyperpigmentation, fibrosis and arm edema.

The mean follow-up was 34.7 (standard deviation (SD): 20.2, min: 2.8, max: 103) months.

The distribution of various categorical variables, in the total sample as well as separately for the subgroups of sequential, sandwich or simultaneous chemo- and radiotherapy, is summarized in table 1.

**Table 1:** Distribution of categorical parameters in patients with sequential, sandwich or simultaneous chemo- and radiotherapy (^a ^done by Pearson x^2^-test)

			**Sequencing of chemo- and radiotherapy**			
		
		**all patients **n = 212	**sequential **n = 86	**sandwich **n = 70	**simultaneous **n = 56	p^a ^(two-sided)
**Age **n (%)	age ≤ 50	41 (19.3)	14 (16.3)	15 (21.4)	12 (21.4)	0.7
	age > 50	171 (80.7)	72 (83.7)	55 (78.6)	44 (78.6)	
**histological type **n (%)	ductal-invasiv	158 (74.5)	65 (79.3)	48 (68.6)	45 (80.4)	0.7
	ductal-lobular	35 (16.5)	14 (17.1)	14 20)	7 (12.5)	
	other	19 (9)	7 (8.1)	8 (11.4)	4 (7.1)	
**pT **n (%)	pT1/2	123 (58)	53 (61.6)	40 (57.1)	30 (53.6)	0.4
	pT3/4	89 (42)	33 (38.4)	30 (42.9)	26 (46.4)	
**pN **n (%)	pN0	48 (22.6)	12 (14)	14 (20)	22 (39.3)	0.004
	pN1	35 (16.5)	18 (20.9)	8 (11.4)	9 (16.1)	
	pN2-3	129 (60.9)	56 (65.1)	48 (68.6)	25 (44.6)	
**LVI **n (%)	yes	77 (36.3)	22 (25.6)	40 (57.1)	15 (26.8)	< 0.0001
	no	135 (63.7)	64 (74.4)	30 (42.9)	41 (73.2)	
**G **n (%)	G1	5 (2.4)	3 (3.5)	1 (1.4)	1 (1.8)	0.2
	G2	89 (42)	36 (41.9)	23 (32.9)	30 (53.6)	
	G3	118 (55.7)	47 (54.7)	46 (65.7)	25 (44.6)	
**ECS **n (%)	yes	110 (51.9)	50 (58.1)	41 (58.6)	19 (33.9)	0.007
	no	102 (48.1)	36 (41.9)	29 (41.4)	37 (66.1)	
**Receptor **(ER and/or PR) n (%)	positive	158 (74.5)	66 (76.7)	53 (75.7)	39 (69.6)	0.6
	negative	54 (25.5)	20 (23.3)	17 (24.3)	17 (30.4)	
**R **n (%)	R0	197 (92.9)	81 (94.2)	64 (91.4)	52 (92.9)	0.9
	R+	15 (7.1)	5 (5.8)	6 (8.6)	4 (7.1)	
**V **n (%)	yes	194 (91.5)	81 (94.2)	60 (85.7)	53 (94.6)	0.1
	no	18 (8.5)	5 (5.8)	10 (14.3)	3 (5.4)	
**RT region **n (%) parasternal fields (in 28.3% created) not separately considered	THW + SIA	148 (69.8)	62 (72.1)	56 (80)	30 (53.6)	0.01
	THW only	17 (8)	6 (7)	6 (8.6)	5 (8.9)	
	SIA only	47 (22.2)	18 (20.9)	8 (11.4)	21 (37.5)	

### Statistical methods

Continuous variables were summarized by minimum, maximum, mean and corresponding standard deviation. Categorical data were condensed by absolute and relative values.

Cross tabulations were created to compare frequency distributions between subgroups (sequential, sandwich, simultaneous). The Pearson x^2^-test was used to assess whether the associations displayed in those cross tabulations are statistically significant.

The observation period started at the time of surgery. Overall survival (OS)- and disease free survival (DFS)- curves were estimated according to the Kaplan-Meier method. The log-rank test was used for global comparison of OS- and DFS-curves between the subgroups according to the following variables: sequencing of chemo- and radiotherapy (SEQ_chemoRT_), age, histological type, pathological tumor stage (pT), pathological nodal stage (pN), lymphovascular involvement (LVI), grading (G), receptor status (Rec), extracapsular spread (ECS), resection status (R), vascular involvement (V) and irradiated regions (RTregion). Moreover, multivariate Cox regression analyses were carried out in order to investigate which of these variables are independent prognostic factors with respect to OS and DFS.

To allow for a better interpretability of the results reached in those Cox regression analyses, for each of the two response variables (OS, DFS) only factors showing a p-value of ≤ 0.1 in the corresponding univariate log-rank analysis were incorporated as independent variables in the according Cox regression models. Additionally local recurrence free survival (LRS)- and distant recurrence free survival (DRS)- curves were estimated according to the Kaplan-Meier method and the log-rank test was used for comparison of LRS- and DRS-curves in relation to the sequencing of chemo- and radiotherapy.

The global significance level for all statistical test procedures conducted was chosen as α = 5%. All statistical analyses were conducted in an explorative manner. Thus, with consideration of the explorative character of the analysis, p-values of p ≤ 0.05 can be interpreted as statistically significant test results. All statistical analyses were carried out using the statistical analysis software package SPSS, version 14.0 (SPSS Inc., Chicago, IL, USA).

## Results

The distribution of the patients categorical parameters stratified by the chemotherapy sequence is presented in table 1. Concerning the parameters nodal involvement (pN), lymphovascular invasion (LVI), extracapsular spread (ECS) and extension of the irradiated region (RTregion) the three treatment groups were significantly imbalanced. The other parameters, e.g. age, pathological tumor stage (pT), grading (G) and receptor status were homogeneously distributed. Looking on those two groups with an equally effective chemotherapy (EC, FEC), the SEQ- and SW-group, the sole imbalance was the extension of LVI (57.1 vs. 25.6%, p < 0.0001).

**Figure 1:**
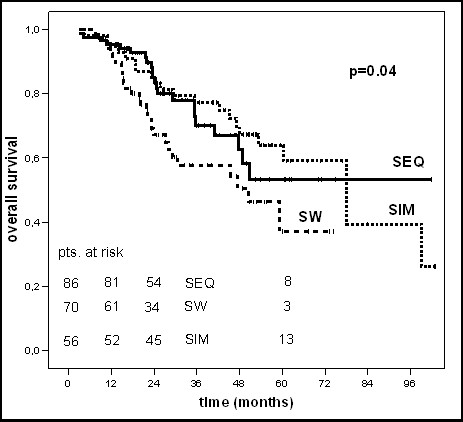
Kaplan-Maier curves of overall survival.

The 5-year overall and disease free survival rate was 53% and 46% for all 212 patients, respectively (table 2). Depending on the sequence of chemotherapy and radiotherapy the 5-year overall or disease free survival rates were 53.2% or 56% (SEQ-group), 38.1% or 32% (SW-group) and 64.2% or 50% (SIM-group), respectively (table 2). There was a significantly difference in 5-year OS and DFS depending on the sequencing of chemo- and radiotherapy in the univariate analysis (p = 0.04 and p = 0.03, log-rank test (table 2 and 3, figure 1).

**Table 2:** 5-year overall- and disease free survival rates depending on subgroups of prognostic factors (* p-values of log-rank tests comparing survival times between subgroups)

			**5-year OS rate**		**5-year DFS rate**	
			
			%	p*	%	p*
**All patients **n (%)		212 (100 %)	53		46	

**Sequencing of chemo- and radiotherapy **n (%)	SEQ	86 (40.6)	53.2	0.04	56	0.03
	SW	70 (33)	38.1		32	
	SIM	56 (26.4)	64.2		50	
**Age **n (%)	age ≤ 50	41 (19.3)	59.4	0.9	52.2	0.6
	age > 50	171 (80.7)	52.2		45.3	
**histological type **n (%)	ductal-invasiv	158 (74.5)	54.2	0.6	47.8	0.6
	ductal-lobular	35 (16.5)	47.6		45.2	
	other	19 (9)	46.8		0	
**pT **n (%)	pT1	33 (15.6)	70.6	0.06	70.8	0.03
	pT2	90 (42.5)	59.8		44.4	
	pT3	41 (19.3)	54.2		45.1	
	pT4	48 (22.6)	31.4		36.8	
**pN **n (%)	pN0	48 (22.6)	65.7	0.1	52.2	0.1
	pN1	35 (16.5)	53.8		53.4	
	pN2-3	129 (60.8)	48.5		42.1	
**LVI **n (%)	yes	77 (36.3)	40.4	0.01	34.9	0.001
	no	135 (63.7)	59.4		52.9	
**G **n (%)	G1	5 (2.4)	33.1	0.04	53.3	0.3
	G2	89 (42)	63.2		49.5	
	G3	118 (55.7)	46.2		44.6	
**ECS **n (%)	yes	110 (51.9)	44.8	0.04	40.2	0.01
	no	102 (48.1)	61.8		52.2	
**Receptor **(ER and/or PR) n (%)	positive	158 (74.5)	56.7	0.07	48.1	0.2
	negative	54 (25.5)	41.3		40.0	
**R **n (%)	R0	197 (92.9)	53.3	0.8	47.3	0.04
	R+	15 (7.1)	56.7		35.0	
**V **n (%)	yes	194 (91.5)	0	0.03	0	0.002
	no	18 (8.5)	55.0		48.3	
**RT region **n (%) parasternal fields (in 28.3% created) not separately considered	THW +SIA	148 (69.8)	52.2	0.4	51.9	0.4
	THW alone	17 (8)	72.8		0	
	SIA alone	47 (22.2)	50.6		36.6	

**Table 3:** 5-year overall, disease free, locoregional recurrence free- and distant recurrence free survival rates depending on the sequencing of chemo- and radiotherapy

			**5-year OS rate**		**5-year DFS rate**		**5-year LRS rate**		**5-year DRS rate**	
			
			%	p*	%	p*	%	p*	%	p*
**All patients **n (%)		212 (100 %)	53		46		75.7		56.4	

**Sequencing of chemo- and radiotherapy **n (%)	SEQ	86 (40.6)	53.2	0.04	56	0.03	88	0.09	60.2	0.1
	SW	70 (33)	38.1		32		57.9		49.8	
	SIM	56 (26.4)	64.2		50		77.4		59.8	

(* p-values of log-rank tests comparing survival times between different sequences)

**Figure 2:**
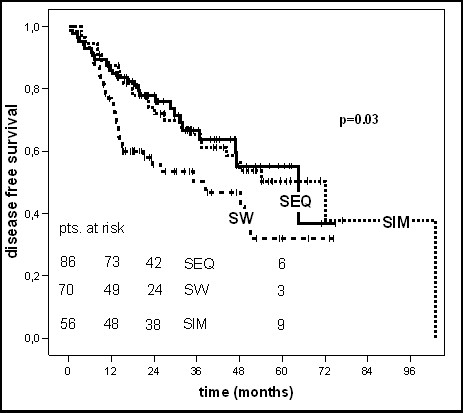
Kaplan-Maier curves of disease free survival.

**Figure 3:**
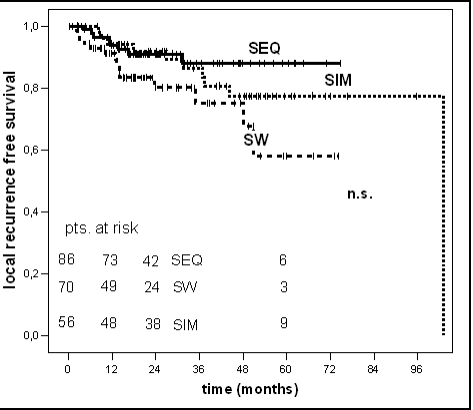
Kaplan-Maier curve of local recurrence free survival.

**Figure 4:**
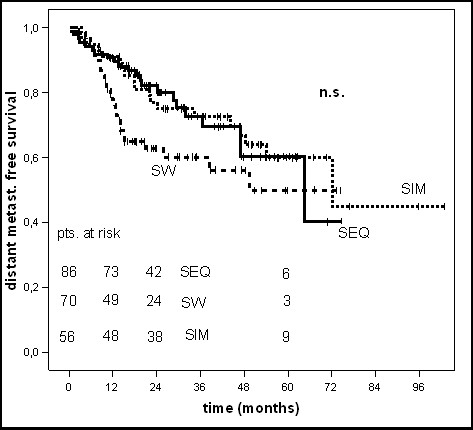
Kaplan-Maier curve of distant recurrence free survival.

Patients in the SEQ-group (n = 86), SW-group (n = 70) and SIM-group (n = 56) had a locoregional and/or distant recurrence in 44%, 68% and 50% within five years, respectively. Patients in the SW-group, which had the highest percentage of LVI (57.1% vs. 25.6% (SEQ-group) vs. 26.8% (SIM-group)), revealed a low 5-year locoregional recurrence free survival rate (LRS, 57.9%) together with a low distant metastases free survival rate (DMS, 49.8%). In dependence on the sequencing of chemo- and radiotherapy no significantly differences could be shown in 5-year locoregional or distant recurrence free survival rates (p = 0.09 and p = 0.1, log-rank test) (table 3 and figures 2, 3, 4).

Moreover the variables LVI, ECS, grading and vascular involvement had significant impact on overall survival times in the univariate analyses (table 2).

In the multivariate analyses (Cox regression) the sequencing of chemo- and radiotherapy showed no independent significant impact on OS (p = 0.2) or DFS (p = 0.4). Only the receptor status revealed an independent influence on OS (p = 0.04), but not on DFS (p = 0.2) (table 4).

**Table 4:** Results (p-values) of multivariate analysis (Cox regression) incorporating various prognostic factors

**Prognostic factors**	**OS**	**DFS**
	**p**	**p**

Sequencing of chemo- and radiotherapy	0.2	0.4
pT	0.07	0.06
pN	0.2	0.7
LVI	0.5	0.2
G	0.1	0.7
ECS	0.7	0.4
Receptor (ER and/or PR)	0.04	0.2
R	0.7	0.4
V	0.08	0.06

## Discussion

The optimal sequencing of adjuvant chemotherapy and radiotherapy in breast cancer patients remains controversial [3]. On the one hand retrospective studies suggest an increase of local recurrence rates when radiotherapy was delayed in favour of finishing chemotherapy [4-6]. On the other hand an increase in distant recurrence was reported when radiotherapy was given first [7-9]. First results from the only existing prospective, randomized trial to proof the question whether the chemotherapy-radiotherapy or radiotherapy-chemotherapy sequence is better was published 1996 by Recht et al. [2]. Patients in the radiotherapy first arm had a lower rate of local recurrence (5% vs. 14%) and a higher 5-year crude rate of distant/regional recurrence (32% vs. 20%) compared to patients in the chemotherapy first arm. The 5-year survival rate of distant recurrence was statistically significant higher in the radiotherapy first arm compared to the rate in the chemotherapy first arm (36% vs. 25%). The updated results with a follow-up of 135 months were published by Bellon et al. in 2005 and showed the loss of significant differences in local or distant recurrence rates between the two treatment arms [1]. Certainly the authors pointed out, that the trial was underpowered to detect small differences in survival times for either sequence. Several other groups found no crucial differences in survival or recurrence rates depending on the sequencing of chemotherapy and radiotherapy, too [7,8,10]. A limiting fact in the assessment of the role of chemotherapy-radiotherapy sequencing may be the use of older chemotherapy regimes, i.e. CMF, which are not appropriate in modern strategies for systemic treatment [11].

Most literature data refer to early breast cancer treated with breast conserving surgery. Data suggesting how to sequence chemotherapy and radiotherapy in a high-risk patient group after mastectomy because of locally or locoregionally advanced breast cancer are very rare. The objective of our analysis was to assess the impact of different sequencing strategies by looking on a patient cohort, which is nevertheless characterized by the process of improvements in chemotherapy strategies.

A total of 212 patients were included in our retrospective analysis. All patients were treated with mastectomy and axillary dissection followed by adjuvant chemotherapy and radiotherapy in the years 1996–2004. In the years 1996 to 1999 CMF, given in 6 cycles, was recommended [12,13] and the mostly used chemotherapy regime in our cohort. Later anthracycline-containing regimes were implemented according to current data [14,15]. The antracycline chemotherapy was used either sequentially or as a sandwich scheme. The decision was based on the estimation of the risk of local or systemic risk. In patients of a predominately local risk, i.e. close surgical margin or peritumoral lymphovascular invasion, a sandwich scheme was preferred. In the other patients with predominately systemic risk, i.e. extensive nodal involvement, the sequential regime was preferred. As from 2003 the sequential regime, given as six cycles FEC prior to radiotherapy was used principally following the current tendency [16] and national recommendations [17].

The 5-year overall (OS) and disease free survival rates (DFS) were 53% and 46%, respectively. The corresponding 5-year locoregional (LRS) and distant recurrence free survival rates (DRS) were 75.6% and 56.4%, respectively, taking all patients into account.

Crucial prognostic factors for OS and DFS are the number of involved axillary lymph nodes [18-21] and lymphatic vessel invasion (LVI) [22,23]. Extracapsular spread in axillary lymph node metastases had likewise a crucial impact on the prognosis [22,24-26].

Our patients belong to a high-risk collective. A total of 60.9 % of our patients had ≥ 4 axillary lymph node metastases, 36.3% had a LVI and 51.9% had an ECS. On average 7 (SD: 7.7) axillary lymph nodes were involved. Regarding the distribution of these prognostic factors OS and DFS in our analysis was comparable to the literature data. Overgaard et al. reported better 5-year OS and DFS rates (72% and 61%) but only 29.1% of the patients had ≥ 4 axillary metastases [27]. Ragaz et al. reported 5-year-OS and DFS rates of 60% and 47% for patients with ≥ 4 axillary lymph node metastases, comparable to our data [28].

In the univariate analysis patients treated with the sandwich regime had a clearly worse 5-year OS and DFS (38.1% and 32%) compared to the sequential (53.2% and 56%) and simultaneous (64.2% and 50%) regime (p = 0.04 for OS and p = 0.03 for DFS, log-rank test). In the multivariate analysis (table 4), conducted to assess the independent influence of various prognostic factors the sequence of chemo- and radiotherapy showed no statistically significant impact on OS (p = 0.2) or DFS (p = 0.4) anymore. The 5-year locoregional- and distant recurrence free survival rates were not statistically significant different in the three groups (p = 0.09 and p = 0.1, log-rank test), too. For OS only the receptor status and for DFS no parameter revealed independent prognostic influence in multivariate Cox regression analysis.

It must be pointed out that the distribution of patient- and tumor-related parameters in our patient groups was unbalanced. In the SW- and SEQ-group significantly more patients had ≥ 4 axillary lymph node metastases (68.6% and 65.1%) compared to the SIM-group (44.6%). A LVI appeared most frequent in the SW-group (57.1%), an ECS in the SW- and SEQ-group (58.6% and 58.1%) (table 1).

The acute and late toxicity data are comparable to the data reported by Markiewicz et al. [29]. The authors analysed the data from 1053 patients concerning the effects of sequence and type of chemotherapy and radiotherapy on cosmesis and complications after breast conserving surgery and found no statistically significant differences.

Because of the imbalances in parameter distributions the results of a retrospective analysis should be interpreted carefully, especially considering the prognostic value of tumor-related factors, like LVI.

In our opinion the use of sequential regimes is state of the art. Simultaneous CMF is feasible concerning the toxicity, but no longer in general use for adjuvant breast cancer therapy [30].

By now the use of anthracyclines is standard in breast cancer chemotherapy and the use of taxanes is clearly increasing, especially in patients with locoregional advanced disease. The simultaneous use of anthracyclines is more than critical because of the known radiosensitizing effect and at least not recommended [11,31-34]. Also paclitaxel simultaneous to radiotherapy leads to higher toxicity, such as pneumonitis and skin reactions [11,35,36].

## Conclusion

So far in the literature no prognostic advantage can be stated for any radio- and chemotherapy sequence in early breast cancer therapy [1,37]. These findings could be confirmed in our analysis in high-risk patients after performing mastectomy. The weakness of retrospective analyses in balancing prognostic factors relativized basically the assessment of the role of sequencing radio- and chemotherapy. In favour to lower toxicity the sequential approach is always recommended. Furthermore radiotherapy should follow chemotherapy according to current guidelines [11,38,39].

## Competing interests

The authors declare that they have no competing interests.

## Authors' contributions

MDP has made substantial contributions to the conception, acquisition of data, analysis and interpretation of data and drafted the manuscript. MP has been involved in acquisition of data and revised the manuscript. BG has been involved in acquisition of data and revised the manuscript SS was involved in statistical analysis and interpretation of data. BA has been involved in acquisition of data and revised the manuscript. MJE has been involved in analysis and interpretation of data, made substantial contributions to the conception and supervised the work. All authors read and approved the final manuscript.

## Pre-publication history

The pre-publication history for this paper can be accessed here:


